# Solubility and pH of bioceramic root canal sealers: A comparative study

**DOI:** 10.4317/jced.54040

**Published:** 2017-10-01

**Authors:** Claudio Poggio, Alberto Dagna, Matteo Ceci, Maria-Vittoria Meravini, Marco Colombo, Giampiero Pietrocola

**Affiliations:** 1 Department of Clinical-Surgical, Diagnostic and Pediatric Sciences, Section of Dentistry, University of Pavia, Italy; 2 Department of Molecular Medicine, Unit of Biochemistry, University of Pavia, Italy

## Abstract

**Background:**

This study compared the solubility and the pH of different root canal sealers *in vitro*.

**Material and Methods:**

BioRoot™RCS, TotalFill BC Sealer, MTA Fillapex, SealapexTM, AH Plus, EasySeal, Pulp Canal Sealer™ and N2 were tested. Similar specimens were prepared using ring molds with an internal diameter of 20 ± 0,1 mm and a height of 1,5 ± 0,1 mm and digitally weighted to register the mass of each specimen before and after immersion in distilled water. Solubility was determined after 24 hours and statistically analysed using a one-way ANOVA test and post-hoc Tukey test. The pH value was measured by a digital pH meter after 3 and 24 hours from manipulation.

**Results:**

BioRoot™RCS and TotalFill BC Sealer showed significantly higher solubility (*P*<0.05). All the remnant root canal sealers fulfilled the requirements of solubility of the International Standard Organization 6876 demonstrating a weight loss of less than 3%. BioRoot™RCS and Totalfill BC Sealer exhibited high alkaline pH over time (*P*<0.05); the alkalinity of the other tested cements was significantly lower.

**Conclusions:**

The prolonged alkalinity of bioceramic sealer matched the increase in solubility. This may encourage their biological and antimicrobial effects, but the ongoing solubility may impact their ability to prevent apical leakage.

** Key words:**pH, root canal sealers, solubility.

## Introduction

Endodontic sealers are used to obtain a stable seal of the root canal systems by the filling of discrepancies between the dentinal wall and gutta-percha (1). Root canal sealers and gutta-percha entomb residual microorganisms, prevent the access of any bacteria from the oral environment and avoid their passage to the periapical tissues, thus aiding the healing of periapical lesions (2). An ideal sealer should offer specific properties (3,4) and insolubility is one of the most desiderable physical property for root canal sealers (5) because it may have a great impact on the success rate of root canal treatment (2). In fact, degradation of the sealer may cause gaps along the sealer/dentin or the sealer/gutta-percha interface which might provide a pathway for microorganisms and their toxic products into periapical tissues (5,6). Low solubility of a root canal sealer has been introduced in 2000 as a requirement in the ANSI/ADA specification No. 57 and in 2001 as a requirement in the International Standards Organization 6876 standard for root canal sealing materials. According to those standards the solubility of a sealer shall not exceed 3% mass fraction after immersion in water for 24 hours (7).

In addition, the pH change of sealers may be related with antimicrobial effects and deposition of mineralized tissue, thus playing a role in the healing process (8). Alkaline pH of root canal sealers could neutralize the lactic acid from osteoclasts and prevent dissolution of mineralized components of teeth (9).

Today different types of endodontic sealers are available: zinc-oxide eugenol (ZnOE), resin-based, calcium hydroxide containing, MTA and bioceramic-based root canal sealers (10). The ZnOE sealers have a long history of successful usage, because of their widely demonstrated positive qualities (4). Calcium hydroxide containing sealers supposedly have antimicrobical effects and biologic properties that stimulate a calcific barrier at the apex (4). Epoxy-based cements are the primarily ones amongst re-sin-based sealers, with many tested properties like antimicrobial action, adhesion to dentin walls, good sealing ability and relative insolubility (4). Because of its favorable biological characteristics, root canal sealers based on mineral trioxide aggregate (MTA) have been introduced (11,). However, the handling characteristics of MTA preclude the use as a sealer without the addition of chemicals that provide sufficient flow (12). Components such as gels or water-soluble polymers have been added to enhance the cement manipulation (12,13). Various studies reported the biocompatibility of MTA endodontic sealers, which may stimulate mineralization and exhibit bioactivity by stimulating hydroxyapatite nucleation (14).

Recently, bioceramic-based sealers containing calcium silicate and/or calcium phosphate attracted considerable attention because of their physical and biological properties such as their alkaline pH, chemical stability within the biological environment, and lack of shrinkage (15) Bioceramic materials contain calcium phosphate which enhances the setting properties of bioceramics and results in a chemical composition and crystalline structure similar to tooth and bone apatite materials, thereby improving sealer-to-root dentin bonding (16).

The purpose of this in vitro study was to evaluate solubility at 24 hours and pH at 3 and 24 hours of new bioceramic-based sealers root canal sealers comparing them to conventional sealers. The null hypothesis tested was that there is no significant difference among root canal sealers tested.

## Material and Methods

Eight different root canal sealers were tested: BioRoot™ RCS, TotalFill BC Sealer, MTA Fillapex, Sealapex™, AH Plus, Easy-Seal, Pulp Canal Sealer™, N2 ( Table 1).

**Table 1:**
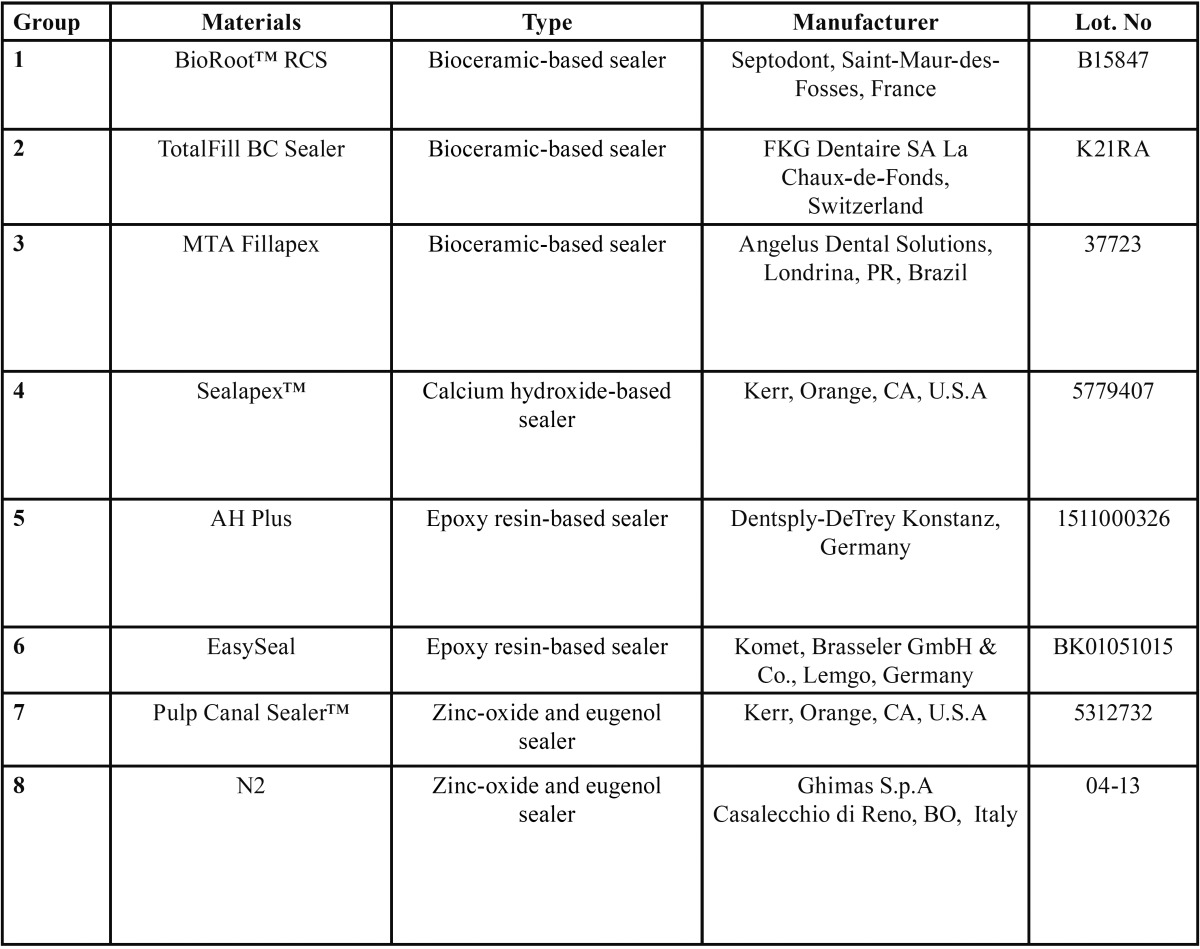
Root canal sealers tested.

-Solubility test

Solubility was determined in accordance with the International Standards Organization (ISO) 6876 method and with the American Dental Association (ADA) specification No. 57. The ISO 6876 standard specifies requirements for “materials used for permanent obturation of the root canal with or without the aid of obturating points”. Stainless steel ring molds with an internal diameter of 20 ± 0,1 mm and a height of 1,5 ± 0,1 mm were used for samples preparation. All moulds were cleaned with acetone in an ultrasound bath for 15 minutes and weighted 3 times before use (accuracy ± 0,0001 g) using a precision balance (Mettler-Toledo, model AE1633, Novate Milanese, Italy). They were then placed on a glass plate, filled to slight excess with the mixed materials and covered with another glass plate under a light pressure in order to remove any exceeding material. All root-canal sealers were mixed and prepared by the same operator in accordance with manufacturer’s instructions. Ten sets of specimens for each material were prepared. All samples set into an incubator at 37˚C and >95% relative humidity (Thermo Fisher Scientific, Waltham, MA, USA) for a period corresponding to three times the setting time. The excess of water was removed with absorbent paper and the samples were weighed 3 times. The average reading was recorded to 3 decimal places. The samples were placed two by two into a Petri dish containing 50 mL of distilled water and transferred into the same incubator at 37˚C and >95% relative humidity for 24 hours. After incubation time, the samples were rinsed with 3 mL of distilled water and the washings were allowed to drain back into the Petri dish. The samples were then discarded, and the Petri dishes were dried in an oven at 105 ˚C for 48 hours (Thermo Fisher Scientific, Waltham, MA, USA), cooled down in the same desiccator and reweighted. The difference between the final mass and the initial mass of the Petri dish divided by the initial dry weight of the sample x 100 correspond to the loss of mass of each specimen express as percentage of solubility (7,16). The solubility test was repeated 2 months after by using the same method (7). Analysis of variance (ANOVA) was applied to determine whether significant differences existed among the groups. For the post-hoc test, the Tukey’s test was used. Significance for all statistical tests was predetermined at *P*<0.05.

-pH measurements

Root canal sealers were mixed and placed onto cylindrical Teflon moulds 2-mm-height and 10-mm-diameter and set into an incubator at 37˚C and >95% relative humidity (Thermo Fisher Scientific, Waltham, MA, USA). Six samples were prepared for each group and were placed into separate vials containing 10 mL of distilled water. The samples were stored at 37°C and pH measurement was performed 3 and 24 hours after incubation. The pH values were measured by a digital pH meter (OrionTM pH Meter 420A, Orion Research Inc., Boston, MA, USA) calibrated with buffer solutions (Orion buffer solution, Perfect buffer 10, Orion Research Inc. [pH=4,01; pH=7,00; pH=10,00]) before each experiment. After removal of the specimens, the container was placed in an orbital shaker (R&D Labs srl, Saronno, Italy) for 5 sec before measuring. The temperature of the room during the test was 25°C. Tukey’s test was applied to determine whether significant differences existed in pH values after 3 hours of incubation. To determine whether time influenced the pH values of the root canal sealers, an analysis of longitudinal data was performed using t-test for paired data (*P*<0.05) between times of incubation (3 and 24 hours).

## Results

-Solubility (%)

The results of solubility test are listed in  Table 2. BioRoot™RCS and TotalFill BC Sealer showed significantly higher (*P* < 0.05) solubility among the tested materials, although the highest solubility percentage was recorded for Totalfill BC Sealer. All remnant root canal sealers (EasySeal; MTA Fillapex; Pulp Canal Sealer™; Sealapex™; AH Plus and N2) fulfilled the requirements of the International Standard Organization 6876 and the ANSI/ADA specification No. 57 for endodontic sealing materials (Chicago, USA, 2000), demonstrating a weight loss of less than 3%. AH Plus provided the lowest solubility, significantly lower (*P* < 0.05) than all the tested sealers. Pulp Canal Sealer™, N2, Sealapex™, EasySeal and MTA Fillapex showed solubility values significantly lower (*P* < 0.05) than BioRoot™RCS and TotalFill BC Sealer but significantly higher (*P* < 0.05) than AH Plus. No significant differences were found between Pulp Canal Sealer™, Sealapex™ and N2. Solubility values in increasing order were: AH Plus < Pulp Canal Sealer™ < N2 < Sealapex™< EasySeal < MTA Fillapex < BioRoot™RCS < TotalFill BC Sealer.

**Table 2:**
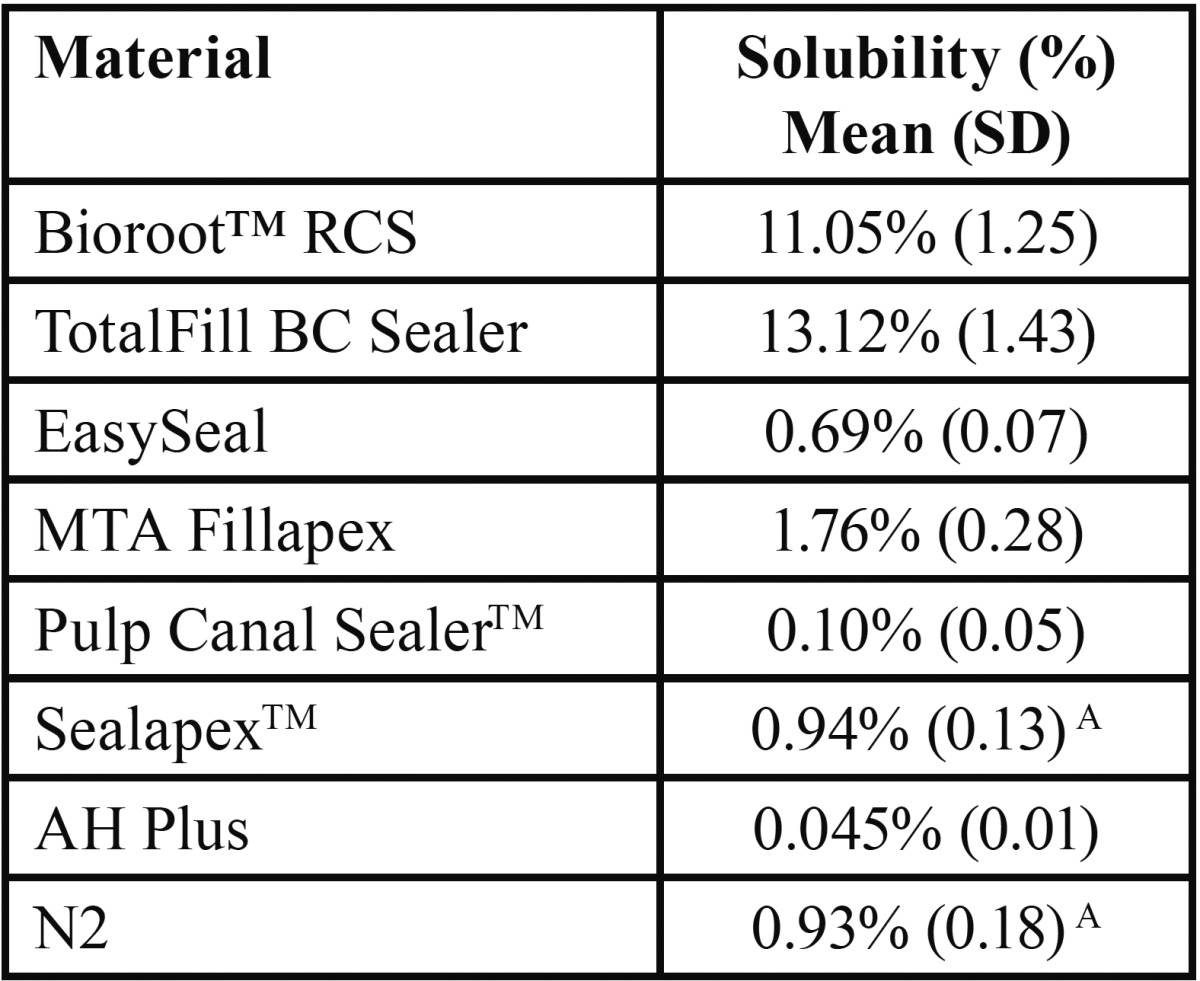
Mean percentage values of solubility and standard deviation (SD) for each material.

-pH changes

The pH mean values of all tested materials at different immersion times (3 and 24 h) are described in  Table 3. BioRoot™RCS, Totalfill BC Sealer and Sealapex™ exhibited high alkaline pH values over time, although the significantly highest alkaline pH score was recorded for TotalFill BC Sealer (*P* < 0.05). No significant variation in pH was observed for Sealapex Root Canal Sealer over time, whereas it was significant for both BioRoot™RCS and TotalFill BC Sealer (*P* < 0.05). Significantly lower (*P*<0.05) was the alkalinity of EasySeal, MTA Fillapex, Pulp Canal Sealer™ and AH Plus than that observed for BioRoot™RCS, TotalFill BC Sealer and Sealapex™. MTA Fillapex exhibited initial neutral pH (7.68) followed by a weak alkaline pH (8.02). Pulp Canal Sealer™ and AH Plus had initial weak alkaline pH (8.0) followed by neutral pH (~7.6). N2 exhibited initial neutral pH (~7.1) followed by final weak acidic pH (~6.98).

**Table 3:**
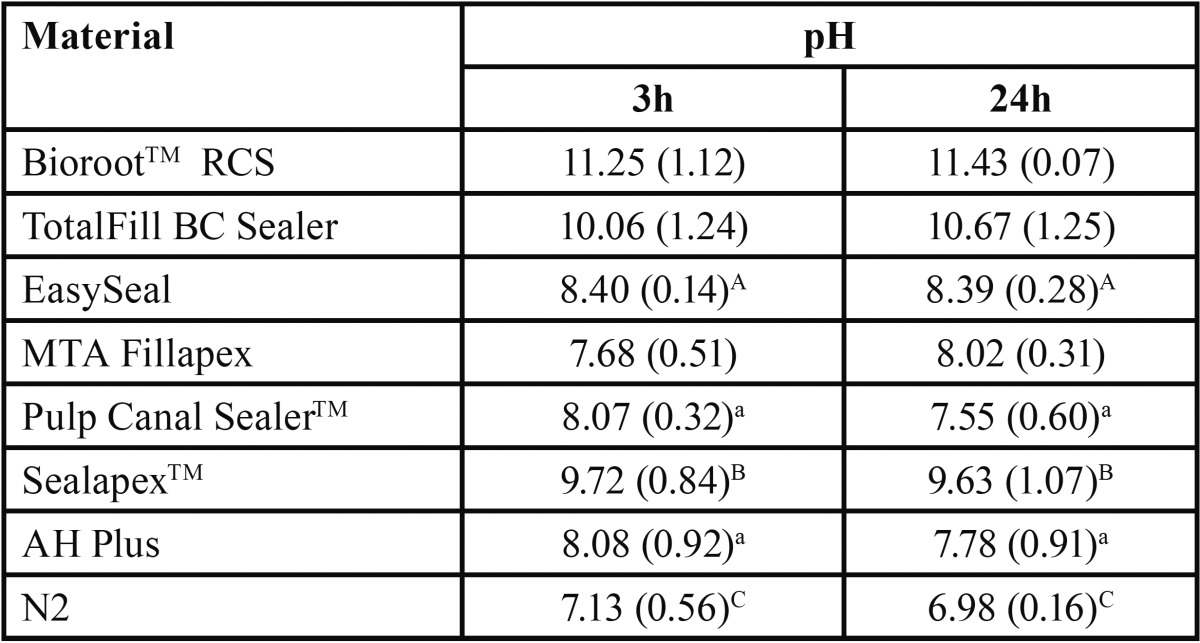
Mean pH values and standard deviation (SD) for each the tested materials at 3 and 24 after incubation.

## Discussion

Root canal sealers have to provide an apical seal avoiding the leakage of irritants and pathogens from the root canal system into the periradicular tissues (4). Solubility is an important factor in assessing the suitability of dental materials in dentistry: it’s defined as the ability of a substance to dissolve in another and it’s expressed as the concentration of the saturated solution of the former in the latter (17). The solubility of the root canal sealers shall not exceed 3% mass fraction after immersion in water for 24 hours, when determined in accordance to the International Standards Organization 6876 standard or ANSI/ADA Specification No. 57. The solubility test performed in the present study fulfilled these standards. This kind of test is significant because root canal sealers can get direct contact with periapical tissues fluids in apical region (18). MTA-based and bioceramic sealers have been introduced in endodontics for advantages like their biocompatibility that prevents rejection by the surrounding tissues and improved sealer-to-root dentin bonding, due to the deposition of bone apatite materials (19).

The null hypothesis of this study was rejected: a significant difference in solubility percentages among the root canal sealers tested has been shown. Furthermore, the findings of this study demonstrated that BioRoot™RCS and TotalFill BC Sealer showed significantly higher solubility among the tested materials with a weight loss higher than 3%.

Many studies investigated the solubility of root canal sealers (2). ZnOE sealers are generally related with a certain degree of weight loss after storage in water, varying approximately from less than 1% to 7% (18). Calcium hydroxide-containing sealers are also believed to be soluble over time (18). Epoxy resin-based sealers have a relatively low solubility in water (18). MTA-based sealers have been reported to fulfill the requirements of the International Standard Organization 6876, demonstrating a weight loss of less than 3% (19).

In the present study BioRoot™RCS and TotalFill BC Sealer showed significantly higher solubility among the tested materials and they reported a weight loss higher than 3%. This is in agreement with a study by Borges *et al.* (20), which demonstrated that solubility of bioceramic sealer iRoot SP didn’t meet ANSI/ADA requirements: high solubility is the result of hydrophilic nanosized particles, which increases their surface area and allows more liquid molecules to come into contact with the sealer. However, Literature contains conflicting results: Viapiana *et al.* (1) found high solubility of MTA-Fillapex, while Zhou *et al.* (10) reported that solubility of the biocearmic sealer EndoSequence BC is consistent with ISO 6876/2001. The discrepancy between the results of these Authors may be attributed to variations in the methods used to dry the samples after having subjected them to solubility testing. The freshly published article by Lee *et al.* (20) showed that Endosequence BC Sealer and MTA Fillapex were not set in humid incubator condition even after one month, so non-complete setting of these root canal sealers should be another reason for higher solubility. Lee *et al.* (21) concluded that both BC Sealer and MTA Fillapex are showed to not fulfill the required chemical and physical properties as ideal root canal sealers.

An alkaline pH may contribute to osteogenic potential, biocompatibility, and antibacterial ability of root canal sealers (8,10,21). The calcium hydroxide sealer tested (Sealapex™) demonstrated the higher alkalinity (pH > 9), epoxy resin-based sealers tested (EasySeal and AH plus) showed a fair alkalinity (followed by a neutral pH at 24 hrs for AH Plus), the ZnOE sealers reported an initial weak alkaline pH (8.0) followed by a neutral pH (~7.6) for Pulp Canal Sealer™ and an initial neutral pH (~7.1) that followed by a final weak acidic pH (~6.98) for N2.

Recent studies indicated that the resin-based sealers like AH Plus are characterized by a slightly neutral pH and low solubility (22). This concept was confirmed by Faria-Júnior *et al.* (23): the neutral pH and its low solubility may reduce the antibacterial activity of the sealer.

The bioceramic-based sealers (BioRoot™RCS and Totalfill BC Sealer) exhibited high alkaline pH over time, significantly lower than the other tested materials. MTA fillapex reported an initial neutral pH (7.68) that was followed by a weak alkaline pH (8.02). Various studies supported these findings about bioceramic-based sealers: their pH is ranging between 10-12 for some weeks after setting (10,22). Silva *et al.* (24) showed that the initial pH of MTA-Fillapex was few alkaline (pH = 9.3) and gradually declined over time to 7.76 after one week.

A strong alkaline pH is supposed to encourage a prolonged setting time and a long-lasting antibacterial effect that eliminates the residual microorganisms survived along dentinal walls. Silva *et al.* (24) suggested that MTA-Fillapex, due to high alkalinity, is able to release hydroxyl ions, thereby causing a high Ca2+ ion release. The alkaline behaviour could contribute to hard tissue formation by activating alkaline phosphatase, neutralize the lactic acid from osteoclasts and prevent dissolution of mineralized components of teeth, prevent the bone destruction and allow tissue repair with hydroxyapatite formation (25). In Lee *et al.* study (21) the pH value of three different bioceramic-based root canal sealers remained significantly higher than that of epoxy re-sin-based sealers for 24 hours, with the highest alkaline pH measured from BC Sealer for the entire period of evaluation.

## Conclusions

Based on the present results, the tested root canal sealers are showed to fulfill the required solubility properties, except the Bio-Root™RCS and TotalFill BC Sealer. Even if pH values may encourage their biological and antimicrobial behaviour over time, the BioRoot™RCS and TotalFill BC Sealer should be improved to reduce their solubility and to increase their ability to prevent apical leakage. Further clinical trial tests and long term follow-up studies would be highly valuable to evaluate the bioceramic sealers’ clinical performances.
